# Documented diabetes care among older people receiving home care services: a cross‐sectional study

**DOI:** 10.1186/s12902-021-00713-w

**Published:** 2021-03-10

**Authors:** Lovise S. Heimro, Monica Hermann, Therese Thuen Davies, Anne Haugstvedt, Johannes Haltbakk, Marit Graue

**Affiliations:** 1 Dept. of Health and Caring Sciences, Western Norway University of Applied Sciences, Stord, Norway; 2 Dept. of Health and Caring Sciences, Western Norway University of Applied Sciences, Bergen, Norway

**Keywords:** Diabetes, Home care services, Documentation, Guideline recommendations

## Abstract

**Background:**

Home care services plays an important role in diabetes management, and to enable older adults remain home-dwellers. Adequate follow-up and systematic nursing documentation are necessary elements in high quality diabetes care. Therefore, the purpose of this study was to examine the diabetes treatment and management for older persons with diabetes receiving home care services.

**Methods:**

A cross-sectional study was used to assess the diabetes treatment and management in a Norwegian municipality. Demographic (age, sex, living situation) and clinical data (diabetes diagnose, type of glucose lowering treatment, diabetes-related comorbidities, functional status) were collected from electronic home care records. Also, information on diabetes management; i.e. follow-up routines on glycated haemoglobin (HbA_1c_), self-monitoring of blood glucose, insulin administration and risk factors (blood pressure, body mass index and nutritional status) were registered. HbA_1c_ was measured upon inclusion. Descriptive and inferential statistics were applied in the data analysis.

**Results:**

A total of 92 home care records from older home-dwelling persons with diabetes, aged 66–99 years were assessed. Only 52 (57 %) of the individuals had the diabetes diagnosis documented in the home care record. A routine for self-monitoring of blood glucose was documented for 27 (29 %) of the individuals. Only 2 (2 %) had individual target for HbA_1c_ documented and only 3 (3 %) had a documented routine for measuring HbA_1c_ as recommended in international guidelines. Among 30 insulin treated older individuals, a description of the insulin regimen lacked in 4 (13 %) of the home care records. Also, documentation on who performed self-monitoring of blood glucose was unclear or lacking for 5 (17 %) individuals.

**Conclusions:**

The study demonstrates lack of documentation in home care records with respect to diagnosis, treatment goals and routines for monitoring of blood glucose, as well as insufficient documentation on responsibilities of diabetes management among older home-dwelling adults living with diabetes. This indicates that home care services may be suboptimal and a potential threat to patient safety.

## Background

In 2019 there were approximately 463 million adults worldwide living with diabetes, and the prevalence rises in parallel with ageing and the number of older people living longer [1]. People with diabetes in general have a risk for developing diabetes related complications, and they are in need of careful follow-up to prevent severe complications such as cardiovascular events, falls, pain, depression, infections and lower limb amputations [2]. Diabetes complications pose a substantial risk to reduced functional status, frailty, and institutionalization, especially at the age of 65 years and above [3, 4]. Frailty is characterized by functional weakening and cognitive disability and influence the older individuals` abilities to self- management [5, 6]. Self-management refers to activities and behaviours individuals undertake to control and treat their condition [7]. To avoid diabetes-related complications, people with diabetes need to undertake continuous self-management, which include both lifestyle interventions (healthy diet, regular physical activity and maintaining a healthy body weight) and pharmacologic interventions (oral medicines, insulin, to control blood glucose level) [1, 2]. Regular blood glucose monitoring is required to effectively manage diabetes [1, 2]. Blood glucose monitoring, recommended for anyone treated with insulin, is a complex task which takes place in the home [2, 8]. Several of the self-management tasks may be challenging to carry out by frail, older individuals, and thus, older home-dwellers with diabetes often need assistance from their next-of-kins or professional health care providers to achieve proper self- management [9].

Enabling older people to remain home-dwelling has become a political priority in many countries [10, 11]. In Norway, more than 10 % of individuals above 80 years receive home care services, and the prevalence of diabetes among individuals aged 65 years and older receiving home care is shown to be 24 % [12]. In Norway, home care services are delivered mainly by the public health service system as a public right for all citizens, organized by the municipalities [13]. In the organization and provision of care, documentation of planned and delivered care is crucial for the quality of home care services.

Evidence-based guidelines recommend HbA_1c_ between 53 mmol/mol (7 %) and 69 mmol/mol (8.5 %) for older adults with diabetes, and emphasize that diabetes related adverse events can be reduced if these recommendations are followed [1, 2, 8]. However, both national and international guidelines emphasize the importance of individualised goals and treatment plans [2, 8]. In addition to individual treatment goals for HbA_1C,_ individualised routines for capillary blood glucose measurements and routines for regular assessments of blood pressure and body mass index (BMI) is recommended in diabetes-guidelines [2, 8].

Self-management is important for people with diabetes in everyday life [14], and reduced ability for self-care makes home care receivers with diabetes at increased risk of adverse diabetes related events [1, 15–17]. As symptoms of hyper- and hypoglycaemia can be atypical in people from the age of 65 years and above, such symptoms can be interpreted as normal geriatric symptoms and therefore be ignored in older people with diabetes [18]. To ensure adequate follow-up, systematic and structured nursing documentation is necessary for continuity of care [19, 20]. A previous study by Gershater et al. [21] found the home care records insufficient regarding documentation of blood glucose and metabolic control in home care services in a Swedish municipality. Gershater et al. [21] showed that a substantial part of people with diabetes had no documented plan for monitoring, evaluation, and diabetes follow-up.

On this basis, the aim of this study was to examine diabetes treatment and management for older people with diabetes receiving home care services in a Norwegian municipality, as documented in electronic home care records. With respect to routines, delivery, and monitoring of care, we investigated what information was documented in electronic home care records, and further evaluated to what degree the documentation of these aspects was in accordance with guideline recommendations.

## Methods

### Design and setting

A cross-sectional study was designed to collect information from electronic home care records of older people (≥ 65 years) with diabetes in home care services in a Norwegian municipality.

### Participants

The sample was recruited from the entire population of individuals aged 65 years and older receiving home care services in a municipality in Western Norway between May 2014 and March 2015, as described by Davies et al. [12]. The age of 65 was chosen as cut off as 65 years and above is the Norwegian Government`s definition of older people, which also corresponds with the retirement age for most people in Norway [22]. From a total of 3666 individuals, a sample of 1100 older individuals were randomly selected. Individuals in terminal/palliative care, with serious medical conditions or severe cognitive impairment were excluded (*N* = 423). Of the remaining 677 persons, altogether 377 persons gave their consent to participation. Among these, there were 92 (24 %) individuals with diabetes, identified by self-report and/or HbA_1c_ > 6.5 %, which constituted the sample of the present study. Self-reported diabetes was identified using the question ‘do you have, or have you ever had, diabetes?’ This question has shown satisfactory validity and reliability [23].

### Collection of data

Demographic and clinical data, as well as data on diabetes management were collected from existing registrations in electronic home care records. The electronic home care records were manually screened by a trained study nurse, and a standardised check-box form was used to register the data. Data were registered as text units in cases where further explanation of information was needed. The following data was registered: age, sex, living arrangement (alone/with others), type of diabetes (type 1/type 2), type of glucose lowering treatment (insulin/peroral/diet), diabetes-related comorbidities/complications (cardiovascular disease, foot-ulcer, polyneuropathy, nephropathy, retinopathy, hypoglycaemia and hyperglycaemia) and other risk factors (blood pressure and BMI (i.e. registered weight and height). Point of care HbA_1c_ was measured upon inclusion for all participants and obtained by analysing capillary blood samples spectrophotometrically using a DCA VantageTM Analyzer (Siemens Healthcare Diagnostics AS, Oslo, Norway). Data on functional status (degree of difficulty with activities of daily living, ADL) in electronic home care records were based on the *Individual-based Statistics for Nursing and Care Services* (IPLOS) [24]. It focuses on the individuals’ levels of functioning in interaction with their surroundings and is used in home care on a regular basis in Norwegian municipalities to verify need of assistance [24]. The variables are based on the *International Classification and Functioning, Disability and Health* (ICF) manual and represent different degrees of disability with the scores from 1 “no problems” to 5 “not able to” carry out ADL. In the present study the scorings were grouped 1–2 “no/minor problems”, 3 “moderate problems” and 4–5 “major problems/ not able to”. Registered information on nutritional status in electronic home care records was based on *The Mini Nutritional Assessment scale* (MNA), a standardized instrument to assess risk of malnutrition [25].

The following information about diabetes management documented in home care records was registered: values, goals and routines for measuring HbA_1c_, values, routines and responsibility of performing self-monitoring of blood glucose, responsibility for insulin administration and documented causes of home care (help with medicine administration, hygiene or other check-ups). Also, any adverse events recorded in the electronic home care records was registered.

### Data analysis

Data on age is presented as median and range. Other data is presented as numbers and percentages to describe frequencies. Text information on causes of home care, who administers insulin and who performs self-monitoring of blood glucose was categorized and grouped according to similarities. Unadjusted difference in treatment type between persons with HbA_1c_ within or outside recommended range was evaluated by Chi-square test. Logistic regression analysis was performed to examine and adjust for possible covariates (age, sex and living situation (alone or with someone)). Difference in median HbA_1c_ measured upon inclusion in individuals with and without previous HbA_1c_ values registered in their electronic home care records was examined by Mann Whitney U test. Statistical significance was defined as P < 0.05. Statistical analyses were conducted using IBM statistics SPSS 27.

### Ethical considerations

The study was approved by the Regional Committee for Medical and Health Research Ethics (2013/2258/REK vest).Before asking for consent, each participant was informed of the study and the possibility to withdraw the consent at any time. Confidentiality was assured by using identification numbers. No incentives were provided to study participants.

## Results

### Sample characteristics 

Home care records from a total of 92 home-dwelling older people (≥ 65 years) with median age 83 years, were assessed. In total, 67 (62 %) individuals were 80 years or older, 43 (47 %) were men and 54 (59 %) lived alone (Table 1). Data of functional status showed that 60 (65 %) of the participants were medium to majorly impaired (Table 1). Information on the causes of need for home care service showed that 67 (73 %) received assistance with medication administration and 57 (62 %) with personal hygiene (Table 1). Altogether, 68 (74 %) of the participants suffered from a cardiovascular disease and 10 (11 %) had a foot-ulcer. One or more hypoglycaemic events were registered in home care records for 17 (18 %) of the individuals, while totally 36 (39 %) individuals had hyperglycaemia registered in their home care records. A total of 159 adverse events were recorded among the participants within the study period of one year, of which 137 (86 %) were due to medication administration.

**Table 1:** Demographic and clinical data of older home-dwelling people with diabetes (*n*=92)

Variable		Median (range)	
Age		83 (66 – 99)	
		N (%)	
Sex			
Men		43 (47)	
Women		49 (53)	
Type of diabetes			
Type 1		9 (10)	
Type 2		44 (48)	
No information		39 (42)	
Living arrangement			
Live alone		54 (59)	
Live with others (family/relatives)		38 (41)	
Functional status			
Minor problems		32 (35)	
Moderate problems		48 (52)	
Severe problems		12 (13)	
Cause of home nursing^a^			
Medicine administration		67 (73)	
Hygiene		57 (62	
Nutrition		22 (24)	
Observation		29 (32)	
Diabetesrelated comorbidities^a^			
Cardiovascular disease		68 (74)	
Foot – ulcer		10 (11)	
Polyneuropathy		7 (8)	
Nephropathy		5 (5)	
Retinopathy		3 (3)	
Hypoglycaemia		17 (18)	
Hyperglycaemia		36 (39)	

^a^ Due to multiple registrations pr. individual the total percentage exceeds 100%

### Documentation of diabetes treatment and management in electronic home care records

Only 52 (57 %) of the individuals had their diabetes diagnosis documented in the home care records, of which 44 individuals (48 %) had type 2 diabetes and nine (10 %) had type 1 diabetes. The remaining 39 individuals (42 %) did not have a diabetes diagnosis registered (Table 1), which included 15 individuals treated with diabetes medication (Table 2). No information on diabetes treatment was found in the home care records for 36 (39 %) of the individuals. The remaining individuals were treated with insulin (n = 30), glucose-lowering per oral medication (*n* = 23) or diet only (*n* = 3) (Table 2).

**Table 2:** Information about diabetes diagnosis and diabetes management documented in home care nursing records (*n* = 92).

Variable	n (%)
Diabetes diagnosis	52 (57)
Information on treatment Diet only Tablet only Insulin treated No information	3 (3) 23 (25) 30 (33) 36 (39)
Goal for HbA_1c_-value	2 (2)
Routine for measuring HbA_1c_	3 (3)
HbA_1c_ value	43(47)
Routine for self-monitoring of blood glucose	27 (29)
Values of self-glucose monitoring	55 (60)
Who performs self-monitoring of blood glucose? Patient Family/relatives Home nursing staff Unclear/ no information	14 1 21 56
Blood pressure	34 (37)
Body mass index (BMI)^a^	63 (68)
Nutritional status	58 (63)

^a^ Information on BMI recorded in electronic home care record or available from registered data (i.e. both height and weight registered)

Values of self- monitoring of blood glucose was available in 55 (60 %) of the home care records (Table 2). Based on the documentation, home care staff assisted in self- monitoring for 21 (23 %) of the individuals, while 14 (15 %) of the older persons with diabetes monitored blood glucose themselves. Among the 30 insulin treated individuals, documentation on insulin regimen was lacking in 4 (13 %) home care records (Table 3). Documentation showed that home care staff was responsible for the administration of insulin for 14 (47 %) of the older individuals receiving home care services, while 10 (33 %) persons administered the insulin themselves (Table 3). In 6 (20 %) of the home care records, documentation on who administers insulin was unclear or lacking. Information on who performs monitoring of blood glucose was unclear or lacking for 5 (17 %) of the insulin users.

**Table 3:** Information on insulin administration and self-monitoring of blood glucose in insulin-users (*n*=30)

Variable	n (%)
Responsible for insulin administration	
Person with diabetes	10 (33)
Home nursing staff	14 (47)
Unclear/ no information	6 (20)
Insulin regimen:	
Yes	26 (87)
No	4 (13)
Goal for HbA_1c_ value:	
Yes	2 (7)
No	28 (93)
Routine for measuring HbA_1c_:	
Yes	3 (10)
No	27 (90)
HbA_1c_ value:	
Yes	19 (63)
No	11 (37)
Routine for self-monitoring of blood glucose:	
Yes	21 (70)
No	9 (30)
Values from self-monitoring of blood glucose:	
Yes	28 (93)
No	2 (7)
Who performs self-monitoring of blood glucose?	
Person with diabetes	9 (30)
Home nursing staff	16 (53)
Unclear/ lacking	5 (17)

### Documentation of individualised routines and treatment goals

**Fig. 1:**
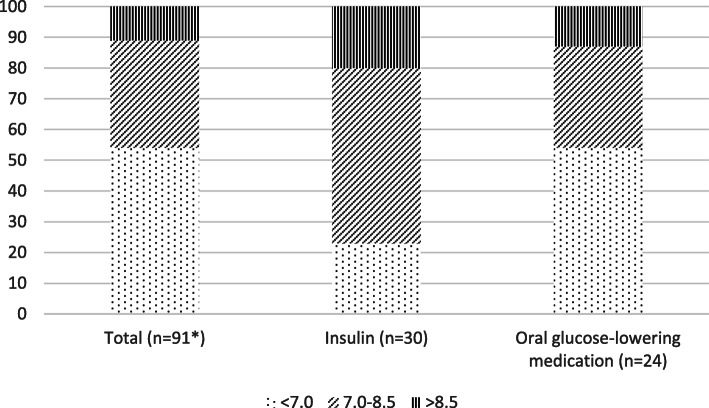
HbA1c-values at the time of inclusion in the total sample, insulin users and users of oral glucose lowering medication

HbA_1c_ values from the last 12 months prior to data collection were found in the home care record for 43 participants (47 %), and only 2 (2 %) had registered individual targets for HbA_1c_ value while only 3 (3 %) had a documented routine for measuring HbA_1c_ (Table 2) as recommended in diabetes guidelines [2, 8]. Current HbA_1c_ values from blood samples withdrawn upon inclusion in the study, showed that 50 (54 %) of the participants had HbA_1c_ levels lower than 53 mmol/mol (7 %) (Fig. 1). The proportion of individuals with HbA_1c_ values within the recommended range were significantly higher in insulin users compared with individuals not using insulin (*P* < 0.05) (Fig. 1). No significant difference in median HbA_1c_ values measured at the time of inclusion was found in individuals with previous registrations in their electronic home care records versus individuals without registered HbA_1c_ values (7.3 vs. 6.9, *P* > 0.1). Among individuals treated with insulin (*n* = 30), values from self-monitoring of blood glucose were registered in 28/30 home care records although a documented routine for self- monitoring lacked in 9/30 (30 %) of the records (Table 3).

Values (height and weight) for estimation of BMI were available in 63 (68 %) of the records (Table 2). The remaining records lacked information on weight, height or both. Blood pressure was registered in only 34 (37 %) and nutritional status in 58 (63 %) of the home care records (Table 2).

## Discussion

The aim of this study was to explore what information was documented with regards to diabetes treatment and management in home care records for older people living at home, and also to assess if planning and delivery of diabetes care was in accordance with international guidelines. We found documentation in home care services to be insufficient with regards to diagnosis, individual treatment goals and routines for measuring of blood glucose. In addition, information on routines and administration of insulin and self-monitoring blood glucose was unclear.

In order to meet the government’s political priority to enable older people to remain in their homes for as long as possible, home care staff plays an important role in the follow up of their various chronic health conditions. In order to provide adequate home care services to older home-dwelling people with diabetes, it is crucial to have adequate information about individual health status, including diagnosis, plan, delivery and monitoring of care and follow-up. The present study revealed lack of documentation of diabetes diagnosis in electronic home care records for almost half of the study population. Similar findings have been observed for Norwegian nursing home residents [26] and home care services in Sweden [21]. We also observed substantial lack of documentation routines on follow-up of HbA_1c_ and self-monitoring of blood glucose. More than half of the home care records in our study lacked values for HbA_1c_ while individual goals for HbA_1c_ was registered in only 2 % of the home care records. According to evidence-based guidelines, severe diabetes related events can be reduced by management of hyper- and hypoglycemia and follow-up goals for HbA_1c_ [1, 2, 8]. Guidelines recommend HbA_1c_ testing in all persons with diabetes, both as an initial assessment and part of continuing care. Measurement approximately every 3 months is recommended to determine whether patient’s glycaemic targets have been reached. The frequency of HbA_1c_ testing should, however, be individualized dependent on the clinical situation [2, 8]. In addition to measurement of HbA_1c_-levels, self-monitoring of blood glucose is in general recommended in the treatment for most people with diabetes, to evaluate their own response to therapy, assess whether glycaemic targets are being achieved, and prevent hypoglycaemia [2, 8]. In the present study, only 3 % of the individuals had documented routines for measuring blood glucose. Also, information on who administered self- monitoring of blood glucose was unclear or lacking for more than half of the home-dwelling people with diabetes. Despite of being recommended for anyone treated with insulin [2, 8] this information was unclear or lacking for 17 % of the insulin-users as well. The lack of documentation of follow-up of blood glucose observed in the present study indicates insufficient follow-up of diabetes in older home-dwelling individuals.

To enable older people with diabetes to live at home as long as possible, home care services play a crucial role to prevent diabetes related complications and to modify risk factors, including hyperglycaemia. As people with diabetes have an increased incidence of hypertension and are at increased risk for cardiovascular events, follow up of blood pressure and BMI is recommended according to guidelines [2]. This is especially relevant in people with type 2 diabetes, where there is often an accumulation of risk factors of cardiovascular disease [2]. In the present study, follow-up of these risk factors was limited. Values on blood pressure were documented in less than half (37 %) of the home care records. In addition, one-third of the home care records lacked information on BMI. The lack of information on follow-up of risk factors indicates that risk factors of cardiovascular disease is inadequately assessed and followed-up in home care.

Organization of home care services in Norway include a larger number of health care workers involved in daily and long-term care. Therefore, it is crucial to ensure that structured and well- informed documentation is available to ensure good information-flow between home care staff involved in the follow-up of the individual with diabetes. In Norway, the general practitioner (GP) is responsible for medical treatment in home-dwelling people and good collaboration between home care staff and GP is therefore essential to ensure that adequate diabetes treatment and follow-up is being conducted. Also, next-of-kins may be involved in the administration of medication and monitoring of blood glucose, although, according to data from this study, family members seemed to have limited responsibility related to diabetes management, as their involvement was documented in only 1 % of the records. The low number of next-of-kins formally involved in diabetes management of home-dwelling individuals with diabetes may be due to their experience of strain when older people with diabetes have impaired health [17]. Findings from a study by Bendixen et al. [27] shows that family members rely on good information and interaction with home care staff in order to experience safety. Messages may be written on private paper in homes as part of the communication between relatives and home care services, and information may also be undocumented [28] which will further affect the continuity of care for older people with diabetes living at home. In older people with diabetes, the aging process may often impact their ability to carry out self-care [29]. In our study, 65 % of the participants had moderate to severe problems related to functional status, and three out of four (73 %) had administration of medication as a central cause of their need for home care services. Nevertheless, one third of those treated with insulin were responsible for measuring blood glucose and administering insulin themselves. Functional status (ADL- score) was similar in those performing self-management tasks themselves and those with help from home care services (data not shown). Moreover, in 39 % of the home care records, including 20 % of the insulin users, we found no information on who was responsible for medication administration and measuring blood glucose.

Lack of information in home care records on the responsibilities related to diabetes management may indicate inadequate organization or inadequate communication between home care service, the individual with diabetes and their next-of-kins. Lack of communication has previously been shown to threaten patient safety [17].

### Strengths and limitations

The results of this study describe documentation routines from home care practice in only one of Norwegians municipalities, and the sample of 92 people can be considered to be a small selection. However, the diabetes diagnosis was verified by a HbA_1c_ blood sample test in all participants. Among the participants enrolled in the study, a total of 14 % were unaware of having the diagnosis. Moreover, the results report planning and delivery of care for a limited period of time, and as such one must show caution in generalizing the results to other municipalities. In addition, it is a limitation that the study only included data from electronic home care records. For some people, individual treatment goals or routines for follow-up of diabetes might be written elsewhere. Also, undocumented routines cannot be excluded. Future studies should attempt to explore factors associated with lack of documentation, as this would be of great interest to improve documentation of diabetes management in older people receiving home care services. Such analyses were not possible in the current study, due to the limited sample size and the lack of permission to collect missing data from alternative sources. The analysis of anonymous data was carried out by researchers who were not employed within the municipality, which can be regarded as a strength for the validity of the results.

## Conclusions

The present study demonstrated lack of documentation with regard to diagnosis, treatment goals and routines for monitoring of blood glucose as well as insufficient documentation on responsibilities of diabetes management among participants. This indicates that diabetes care for older people with diabetes receiving home care services may be suboptimal and a potential threat to patient safety. Efforts to improve documentation routines might be put into practice to ensure that planning and delivery of diabetes care align to current guidelines.

## Data Availability

The dataset is available from the last author, MG (marit.graue@hvl.no) upon a reasonable request.

## Abbreviations

IDF: IDF Diabetes Atlas 9th Edition
ADA: American Diabetes Association
HbA_1C_: Glycated Haemoglobin
BMI: Body Mass Index
GP: General Practitioner
